# 儿童其他医源性免疫缺陷相关淋巴增殖性疾病一例报告并文献复习

**DOI:** 10.3760/cma.j.issn.0253-2727.2023.12.015

**Published:** 2023-12

**Authors:** 慧霞 高, 彦龙 段, 春菊 周, 宁宁 张, 玲 金, 菁 杨, 爽 黄, 梦 张, 永红 张

**Affiliations:** 1 国家儿童医学中心，首都医科大学附属北京儿童医院儿童肿瘤中心肿瘤内科，儿童血液病与肿瘤分子分型北京市重点实验室，儿童肿瘤国家临床重点专科，儿科重大疾病研究教育部重点实验室，北京 100045 Medical Oncology Department, Pediatric Oncology Center, Beijing Children's Hospital, Capital Medical University, National Center for Children's Health, Beijing Key Laboratory of Pediatric Hematology Oncology, National Key Clinical Discipline of Pediatric Oncology, Key Laboratory of Major Diseases in Children, Ministry of Education, Beijing 100045, China; 2 首都医科大学附属北京儿童医院病理科，北京 100045 Department of Pathology, Beijing Children's Hospital, Capital Medical University, Beijing 100045, China; 3 首都医科大学附属北京儿童医院影像科，北京 100045 Department of Imaging, Beijing Children's Hospital, Capital Medical University, Beijing 100045, China

医源性免疫缺陷相关淋巴增殖性疾病指的是异基因造血干细胞移植或器官移植后发生的淋巴增殖性疾病，通常与EBV相关。而其他医源性免疫缺陷相关淋巴增殖性疾病（Other iatrogenic immunodeficiency associated lympholiferative disorders，OIIA-LPDs）则是在原发病接受免疫抑制药物治疗或其他非移植治疗后的患者中发生的继发的淋巴增殖性疾病[1]。本文报道1例儿童T淋巴母细胞淋巴瘤（T-lymphoblastic lymphoma，T-LBL）在维持化疗期间发生的Epstein-Barr病毒（Epstein Barr virus，EBV）相关OIIA-LPDs的临床特征、治疗过程及预后，指出其管理策略与原发淋巴瘤不同，旨在提高对这一疾病的认识。

## 病例资料

患儿，男，5岁11个月，因“间断发热、咳嗽2个月”于2017年12月13日入院。胸部CT（图1A）示右前上纵隔旁不规则占位性病变，纵隔肿物穿刺病理（图2A～C）诊断为非霍奇金淋巴瘤，WHO分型为T-LBL/ALL，St.Jude分期为Ⅳ期。受累部位：颈部双侧、右侧腹股沟、胸膜、胸腺、心包及纵隔淋巴结（最大者9.8 cm×5.9 cm×9.7 cm），左侧髂骨骨髓灶性（PET/CT），无肝肾受累依据。全血及血浆EBV-DNA定量检测阴性，细胞及体液免疫功能正常。按2017儿童淋巴瘤协作组方案-淋巴母细胞淋巴瘤（CNCL-2017-LBL）中危方案对患儿进行序贯化疗，患儿对化疗敏感，化疗4个月（2018年4月1日）中期影像评估可见纵隔瘤灶退缩（图1A）。2018年11月26日强化疗结束后行系统评估：胸部增强CT及PET/CT显示完全缓解，细胞免疫检测示辅助T淋巴细胞绝对值达到正常低限水平，为0.9×10^9^/L。于2018年12月4日进入维持治疗，每日口服6-巯基嘌呤（6-mercaptopurine，6-MP）（50 mg·m^−2^·d^−1^），每周1次肌注甲氨蝶呤（20 mg/m^2^），每月1次静脉推注长春新碱，口服地塞米松每月5 d，定期三联鞘内注射及评估。

**图1:**
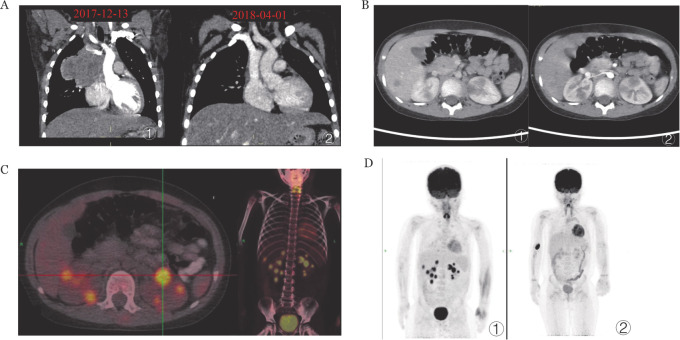
治疗前后病灶影像学变化 A ①初诊时右前上纵隔旁不规则占位病变；②化疗中期评估纵隔瘤灶较前明显退缩； B ①维持治疗1年，肝右叶新发一枚稍低密度结节灶；②左、右肾各新发数枚低回声结节灶； C、D ①PET/CT示肝及双肾多发低密度结节； D ②停化疗后复查PET/CT肝及双肾异常高代谢灶消失

**图2:**
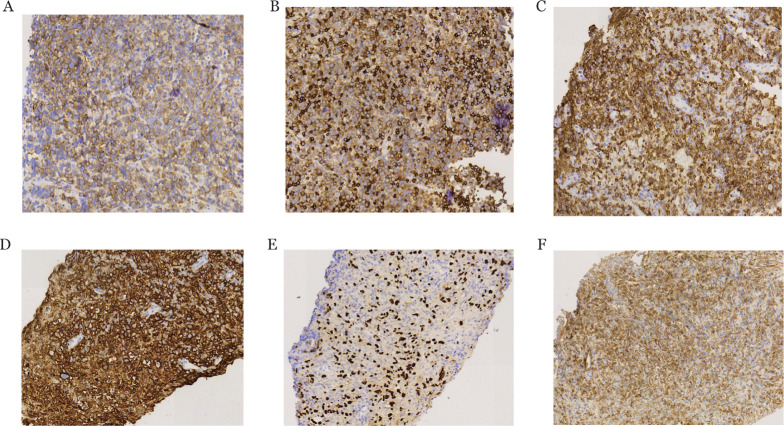
初诊（A～C）及维持治疗1年（D～F）肝脏组织免疫组化染色病理图（×200） A CD99（+）； B CD7（+）； C CD3（+）； D CD20（++++）； E EBER（+）； F CD99（−）

2019年12月24日（化疗2年、维持治疗1年后）患儿再次出现发热症状，予头孢哌酮钠舒巴坦钠、万古霉素及伏利康唑联合抗感染，患儿呼吸道症状缓解，但仍间断中低热，多次病原学检测均阴性，腹部增强CT示肝右叶及双肾等处新发病灶（肝右叶一低回声结节，大小1.2 cm×1.2 cm×1.1 cm；右肾2枚低回声结节，大小分别为0.9 cm×1.0 cm×1.0 cm，1.1 cm×1.1 cm×1.2 cm；左肾2枚低回声结节，大小分别为1.7 cm×1.6 cm×1.3 cm，1.5 cm×1.3 cm×1.3 cm）（图1B）。PET/CT示肝及双肾多发低密度结节，葡萄糖代谢异常增高，锁骨右侧上、纵隔4R区及胸腺部位多发代谢轻度增高淋巴结，最大标准化摄取值（SUVmax）分别为7.3，11.8和2.6（图1C）。同期腰椎穿刺和骨髓病理评估：原发部位淋巴瘤缓解，未见噬血现象。外周血全血EBV-DNA定量2.09×10^4^ 拷贝数/ml，血浆EBV-DNA定量<500 拷贝数/ml；血常规示白细胞减少伴明显的淋巴细胞绝对值减低，最低时达到0.82×10^9^/L。细胞免疫检测示淋巴细胞1.855×10^9^/L；外周血总T淋巴细胞占86％，绝对值为0.775×10^9^/L；总B淋巴细胞占0.9％，绝对值为0.008×10^9^/L；T辅助淋巴细胞占18.5％，绝对值为0.167×10^9^/L；CD4/CD8比例为0.28，见图3。HIV检测阴性。行肝脏结节穿刺，病理光镜下见肝脏及片状坏死，伴大量淋巴细胞浸润、部分细胞体积较大，异型明显，可见核仁及少量胞浆，呈免疫母细胞样或霍奇金细胞样；免疫组化显示CD20（++++）、CD79a（+）、CD30（+）、CD99（−）及Ki-67>50％，原位杂交：EBER（+）（图2D～F），病理诊断EBV阳性大B细胞淋巴瘤（DLBCL）。结合患儿既往诊疗情况及免疫功能提示THL低下，诊断OIIA-LPDs的肿瘤期、T-LBL缓解期。立即停止维持期化疗药物，予利妥昔单抗（375 mg/m^2^）每3周1次，共7个治疗周期。超声监测肝肾结节逐渐减小，3次利妥昔单抗治疗后超声提示肝肾结节消失，复查外周血EBV-DNA载量转阴。2020年5月21日（应用利妥昔单抗5个月）患儿OIIA-LPDs相关治疗结束，未再予原发病T-LBL相关化疗。2020年8月19日复查PET/CT扫描未见病灶，疗效评估为完全缓解（CR）（图1D）。化疗维持期T辅助淋巴细胞明显减少，B辅助淋巴细胞显著缺失，停止维持化疗及利妥昔单抗治疗后淋巴细胞亚群绝对计数恢复正常（图3）。定期行PET/CT、EBV-DNA定量及细胞免疫功能检测。无额外化疗随访至2023年1月，总随访时间5年，OIIA-LPDs后2年8个月，患儿一般情况良好。

**图3:**
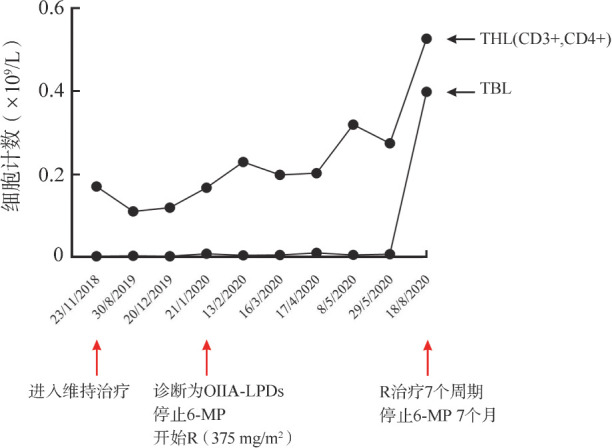
细胞免疫检测淋巴细胞绝对计数趋势 THL：T辅助淋巴细胞；TBL：B辅助淋巴细胞；OIIA-LPDs：其他医源性免疫缺陷相关淋巴增殖性疾病；6-MP：6-巯基嘌呤；R：利妥昔单抗

## 讨论并文献复习

OIIA-LPDs是一类患原发病而长期使用免疫抑制剂导致的淋巴增殖性疾病，组织形态上从多形性细胞增殖到单形性（即淋巴瘤期）。目前修订的世界卫生组织第4版将OIIA-LPDs归类至免疫缺陷相关淋巴组织增殖性疾病[2]。OIIA-LPDs在接受各种免疫抑制剂和免疫调节治疗的成人风湿病患者中有报道[3]–[6]，但只有极少数接受血液肿瘤化疗的儿童病例报告。因该类病例临床较少见，尚未有统一的诊疗指南和共识，极易误诊、漏诊，且其管理治疗策略不同于原发淋巴瘤。

本例患儿病初表现为纵隔占位性病变，组织病理诊断T-LBL明确，无肝肾受累依据，全血及血浆EBV-DNA定量阴性，免疫功能正常。按CNCL-2017-LBL中危方案序贯化疗，瘤灶对化疗敏感，进入维持治疗前系统评估为CR。维持治疗1年，即将结束化疗疗程，患儿PET/CT显示不同于病初的受累部位，肝及双肾多发低密度结节，且肝穿刺组织免疫病理明显不同于病初表现：形态学示肝脏细胞坏死伴多量散在的异型性明显的淋巴细胞浸润，细胞形态偏大。且对比两次病理：病初免疫组化示典型T淋巴母细胞淋巴瘤标志：CD99（+）、CD3（+）、CD4（+）、CD7（+）、CD20（−）、CD30（−）和PAX-5（−），再发时病理淋巴母细胞标志CD99（−）、CD3（−）、CD4（−）、CD7（−），而B细胞标志强阳性［CD20（++++）、PAX-5（+），CD30（散在大细胞+）］，且EBNA2和EBER均呈强阳性，细胞及体液免疫功能检测结果均提示患儿免疫功能严重低下，CD4/CD8比例倒置，外周血辅助T细胞及B淋巴细胞绝对计数均极度低下，且全血EBV-DNA 定量由病初阴性转阳、血浆DNA阴性，提示EBV在细胞内。患儿再发病灶累及多个器官且不同于病初受累部位，免疫组化显示为两种不同起源的淋巴瘤。关于肿瘤维持期宿主免疫指标的报道有限，但就目前已报道的EBV相关的淋巴增殖性疾病都出现在化疗维持期[5],[7]，提示低剂量化疗可能会损害细胞免疫。

OIIA-LPDs的发病机制尚不完全明确，宿主免疫功能低下通常是最关键的致病因素，其他如EB病毒感染、免疫抑制剂的使用、化疗诱导的DNA损伤及宿主遗传易感性等亦在OIIA-LPDs的发生、发展中扮演重要角色[8]。大多数免疫功能正常的人感染EB病毒后处于长期潜伏感染状态，而在自身免疫功能低下的宿主体内，EBV编码产物干扰T细胞的免疫应答，抑制细胞因子活性等来逃避免疫监视从而持续存在，并诱导淋巴瘤的形成[9]。EB病毒编码的潜伏蛋白可损害细胞DNA循环周期起始位点，导致基因组不稳定[10]，同时，EB病毒还可影响B淋巴细胞中相关肿瘤基因（如Bim肿瘤抑制基因、TCL-1癌基因）发挥致肿瘤作用[11]。目前已报道的一例巯嘌呤甲基转移酶（TPMP）缺陷的儿童T-LBL患者在接受6-MP治疗时发展成多灶性EBV相关B细胞淋巴瘤[12]，提示可能与维持药物的累积有关。Elitzur等[13]报告了38年内12家中心合作收治的85例儿童ALL患者治疗后发生非霍奇金淋巴瘤，其中以成熟B淋巴细胞增殖为优势亚型者有56例（66％），这其中又有82％在维持治疗期间或维持治疗后6个月内发展为OIIA-LPDs，表现为与免疫缺陷相关的组织病理学特征，亦提示化疗维持药物暴露的累积或延迟效应与该病相关。当第一种淋巴瘤形成后，一方面肿瘤细胞持续释放细胞因子或慢性刺激信号作用于体内正常的淋巴细胞，加之维持治疗对机体免疫系统的持续损害导致宿主免疫监视能力下降形成免疫抑制微环境，使异常的淋巴细胞不受限制地增殖形成新的恶性克隆[14]–[15]。回顾本例患儿病史发现，在序贯化疗2年的背景下，患儿T细胞功能缺陷及B淋巴细胞增殖失调，与此同时，外周血中检出EBV转阳，进一步干扰T细胞的免疫应答，抑制细胞因子活性等逃避免疫监视从而引起淋巴细胞持续增殖，形成肿瘤。综上，最终考虑诊断OIIA-LPDs（EBV阳性弥漫大B细胞淋巴瘤）。

临床医师对这类肿瘤的认识不足可能会延误诊断。识别OIIA-LPDs的重要意义在于指导治疗，不同于常规原发淋巴瘤继续化疗，及时停用免疫抑制药物是治疗OIIA-LPDs的关键。OIIA-LPDs的发展是在缺乏T细胞监视的情况下B细胞被EBV感染后增殖失调的结果，因此减少进一步的免疫抑制，通过恢复机体原本的细胞免疫来控制疾病。Elitzur等[13]报告了38年内12家中心收治的85例儿童ALL 继发OII-LPDs的治疗，38％的患者接受了全身化疗，其中三分之一采用低剂量、降级化疗方案，而45％的患者接受了利妥昔单抗单药或联合用药。此外，绝大部分接受维持化疗的儿童血液肿瘤患者没有发生OIIA-LPDs，这也提示宿主遗传易感性可能在疾病的发生发展中有一定作用。本例患儿诊断EBV阳性弥漫大B细胞淋巴瘤后，结合临床考虑OII-LPDs诊断，未再采取强化疗，及时停止原有化疗且及时针对B细胞增殖应用了免疫靶向治疗，最终取得了很好的结果。

综上，本例报告强调了临床医师应该提高对血液恶性肿瘤化疗后免疫功能低下时期发生的OIIA-LPDs的认识，及时识别本病及停用免疫抑制药物是治疗的关键。
